# Noninvasive CSF shunt patency evaluation by superb microvascular imaging

**DOI:** 10.1007/s10143-023-02090-5

**Published:** 2023-08-01

**Authors:** D. Putzer, K. Brawanski, M. Verius, H. Oberherber, C. Thome, E. R. Gizewski, H. Gruber

**Affiliations:** 1 Department of Radiology, Medical University Innsbruck, Anichstraße 35, 6020 Innsbruck, Austria; 2 Department of Neurosurgery, Medical University Innsbruck, Anichstraße 35, 6020 Innsbruck, Austria; 3 Canon Medical Systems GmbH, IZ-NÖ Süd, Ricoweg 40, Wiener Neudorf, Austria

**Keywords:** CSF shunt flow evaluation, CSF flow measurement, Shunt diagnostics, Superb microvascular ultrasound, VP shunt dysfunction, VP shunt obstruction, VP shunt sonography

## Abstract

Occlusion of a ventriculoperitoneal shunt system is a potentially life-threatening complication in patients suffering from hydrocephalus. However, there is no imaging established as standard approach in this acute setting. In the present study, we evaluate the use of superb microvascular imaging for investigation of the patency of ventriculoperitoneal shunt systems. Simulation of low flow rates of cerebrospinal fluid through a small diameter CSF shunt system was performed in a dedicated phantom in order to proof the principle of superb microvascular imaging (SMI) being feasible for the measurement of slow CSF flow through the dedicated CSF shunt system. SMI is able to detect low flow rates in CSF shunt systems effectively and fast. Visualization of a Duplex ultrasound flow and Doppler wave pattern in the VP shunt system after the reservoir has been pressed confirms patency. SMI is an effective method for evaluating CSF shunt patency and diagnosing shunt obstruction. This bears the potential to facilitate evaluation of clinically symptomatic VP shunt patients in an acute setting. Further evaluation of ultrasound flow patterns is granted.

**Supplementary Information:**

The online version contains supplementary material available at 10.1007/s10143-023-02090-5.

## Introduction

Ventriculoperitoneal (VP) shunt implantation is the standard treatment for patients suffering from hydrocephalus, a condition of excess accumulation of cerebrospinal fluid (CSF) in the ventricular space. The main reason for hydrocephalus is an obstruction of normal CSF flow, which makes it a serious and life-threatening neurological disorder [1]. First symptoms are heterogeneous and include cognitive decline, incontinence, and reduced vigilance. Subsequently, it can cause severe damage to the brain by increased intracranial pressure and consecutive ischemia.

VP shunt placement is a simple and effective neurosurgical procedure, established in 1960s [2]. The shunt system allows drainage of excess CSF to regulate intracranial pressure by diversion of the fluid in the peritoneal space. Shunt implantation was first performed using thin elastic tubes, which subcutaneously extend to the peritoneal space. Most shunt systems are equipped with a pump reservoir and a valve in order to allow flow control and prevent reflux. Once a patient has a shunt system implanted, he/she usually remains dependent on the system for life.

In case patients with a VP shunt system present with acute symptoms of hydrocephalus in an acute setting, occlusion of the VP shunt system has to be excluded in order to plan further clinical treatment, potentially leading to surgical revision of the VP shunt system. The evaluation of the patient requires high clinical expertise, as acute symptoms are unspecific, including headache and vomiting. Additionally, rapid evaluation is mandatory, as unresolved shunt malfunction can quickly become life-threatening. The diagnosis of shunt obstruction and the localization of the obstruction site are still lacking adequate mechanical instrumentation. Upon hospital admission, patients showing clinical signs of shunt obstruction undergo investigations using ionizing radiation, invasive tests, and open surgical exploration, bearing several risks including the danger of possible brain damage. In the first year following implantation, up to 40% of patients need repeated surgery, and 10% will additionally undergo surgery annually after the first year [3]. Possible reasons for shunt dysfunction include improper shunt placement, disconnection of shunt segments, breakage of the shunt catheter, and infection, component malfunction, and obstruction by accumulation of cellular materials mainly in the ventricular catheter.

The accurate clinical evaluation in patients with symptoms of shunt obstruction is essential, as there are no feasible tests available. Neurosurgeons routinely apply pressure on the silicon membrane of the pump reservoir, which is an integral part of most shunt systems distally to its ventricular part and proximal to its peritoneal part of the shunt system. Simultaneously, the distal end of the catheter is obstructed by manual pressure in a subcutaneous localization. Upon release of the pressure, the reservoir of the system refills in an antegrade manner, which should be visible as the silicon membrane of the pump reservoir causes a small pouch on the skin level. However, this clinical evaluation is not always possible and does not allow any conclusion on the localization of the obstruction.

Ultrasound (US) is an attractive imaging approach in this setting, as it is readily available, not time consuming and can be performed in different locations according to the course of the CSF shunt system. The tubing of CSF shunt systems typically has a lumen diameter of 1 mm and is made of silicone rubber, which is a transparent layer for US, while the inner surface of the shunt systems is not completely smooth, causing disruptions of a potentially laminar flow[4, 5]. Due to the low flow rates that result from the low drainage volume per time unit, the small diameter of CSF shunt systems, and large flow fluctuations, conventional Duplex US systems have a limited sensitivity to detect flow within the catheter, especially as CSF is a clear colorless fluid, which consists of water to 99%. The shunt systems are reported to allow very low flow rates in combination with a high variability of these flow rates in correlation to patient positioning. Drake et al. reported flow rates in CSF shunt systems between 3 and 40 ml/h [6], corresponding to 0.05 ml/min and 0.7 ml/min. In accordance, Kadowaki et al. published data with a physiologic flow rate between 0.01 and 0.1 ml/min [7]. From a technical point of view, modern shunt systems offer programmable, hydrostatic, and flow-controlled valves as well as anti-siphon devices to prevent unphysiological flow rates, upon vertical body positions. In vertical body position, the flow rates observed reach from 93 to 232 ml/h, corresponding to 1.5 to 3.9 ml/min, respectively [8].

Superb microvascular imaging (SMI) is a novel US imaging technique allowing the visualization of low velocity blood flow in small vessels. The essential technical aspect of this new US procedure is based on the fact that noise generated from motion artifacts can be suppressed. The images contain a monochrome color map of blood flow, which is registered onto the B-mode image. We therefore wanted to evaluate the technical feasibility of SMI US visualizing patent flow in VP shunt systems simulating physiologic flow rates in a phantom model.

## Methods

**Fig. 1:**
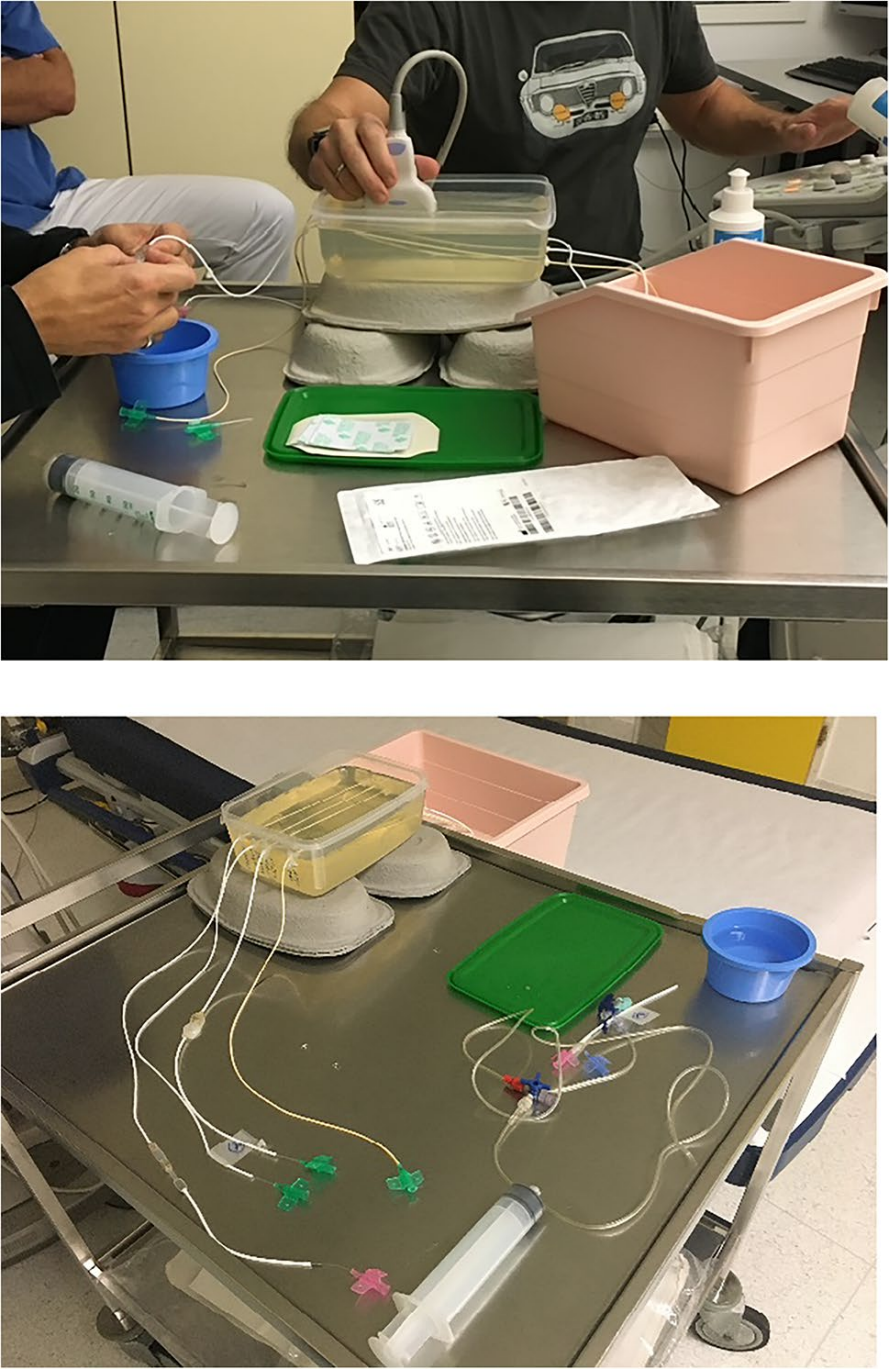
The images show the experimental set up for the simulation of CSF flow through the ventriculoperitoneal shunt system. Three shunt systems were placed in parallel within the phantom, creating a slope inside the phantom for ease of US examination. The fluid was inserted into the shunt system manually using a 20-ml syringe

**Fig. 2:**
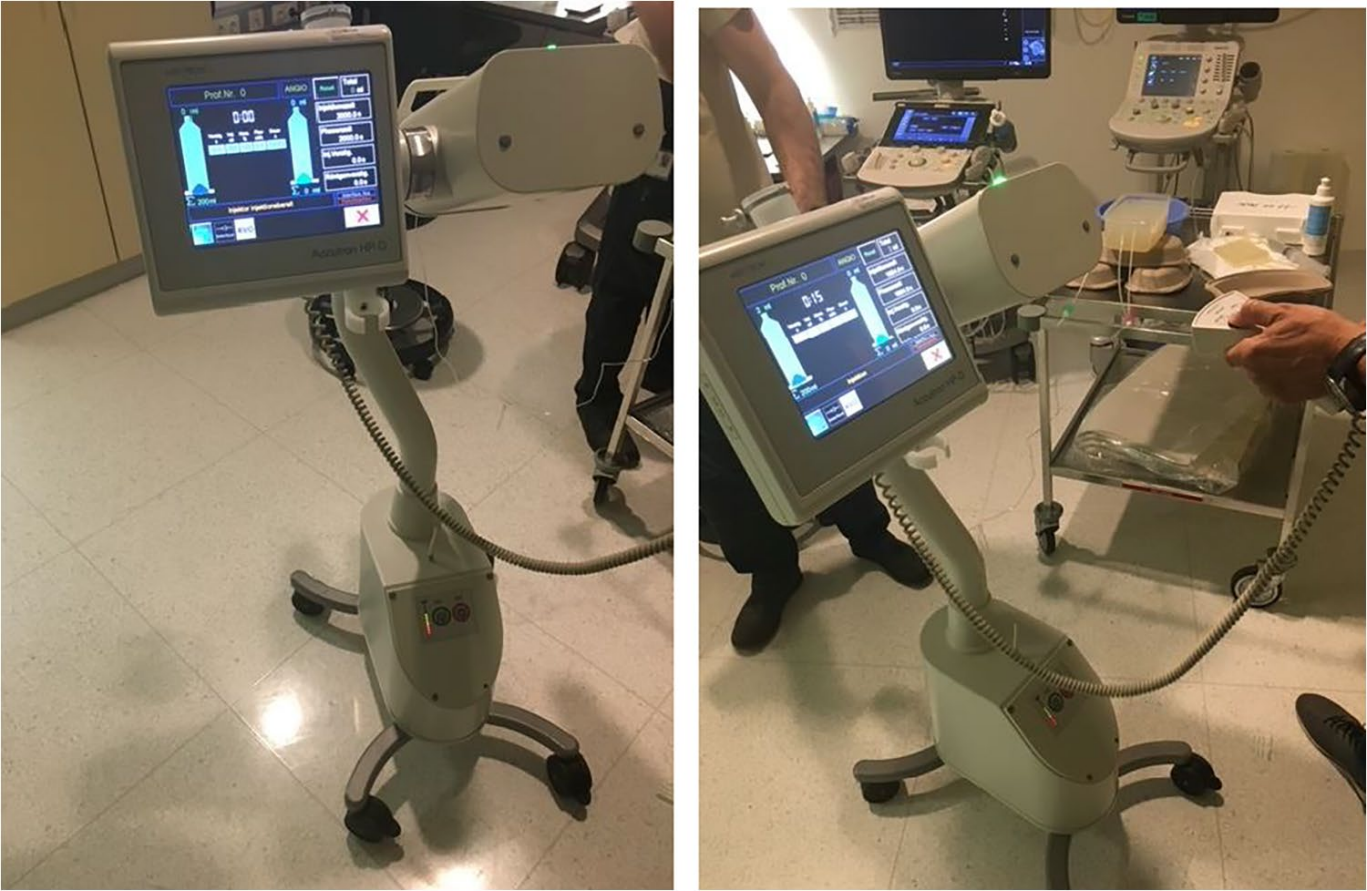
Standardized flow rates inside the VP shunt system phantom were created, using a pressure pump system for angiographic purposes allowing low flow rates

**Fig. 3:**
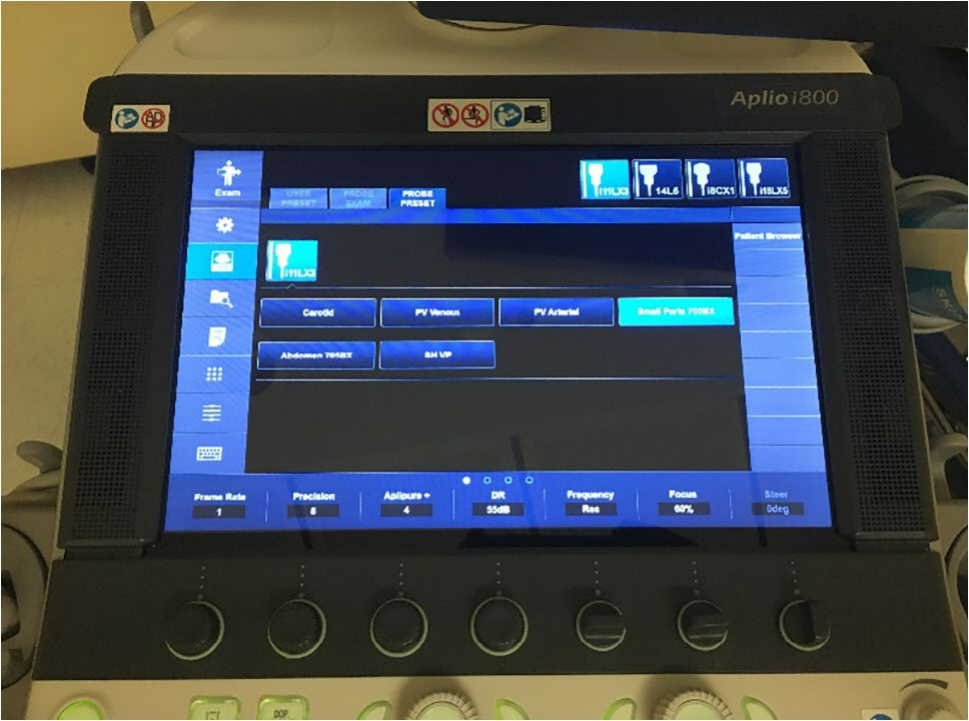
Visualization of the SMI standard imaging protocol on the Aplio i800 US device

**Fig. 4:**
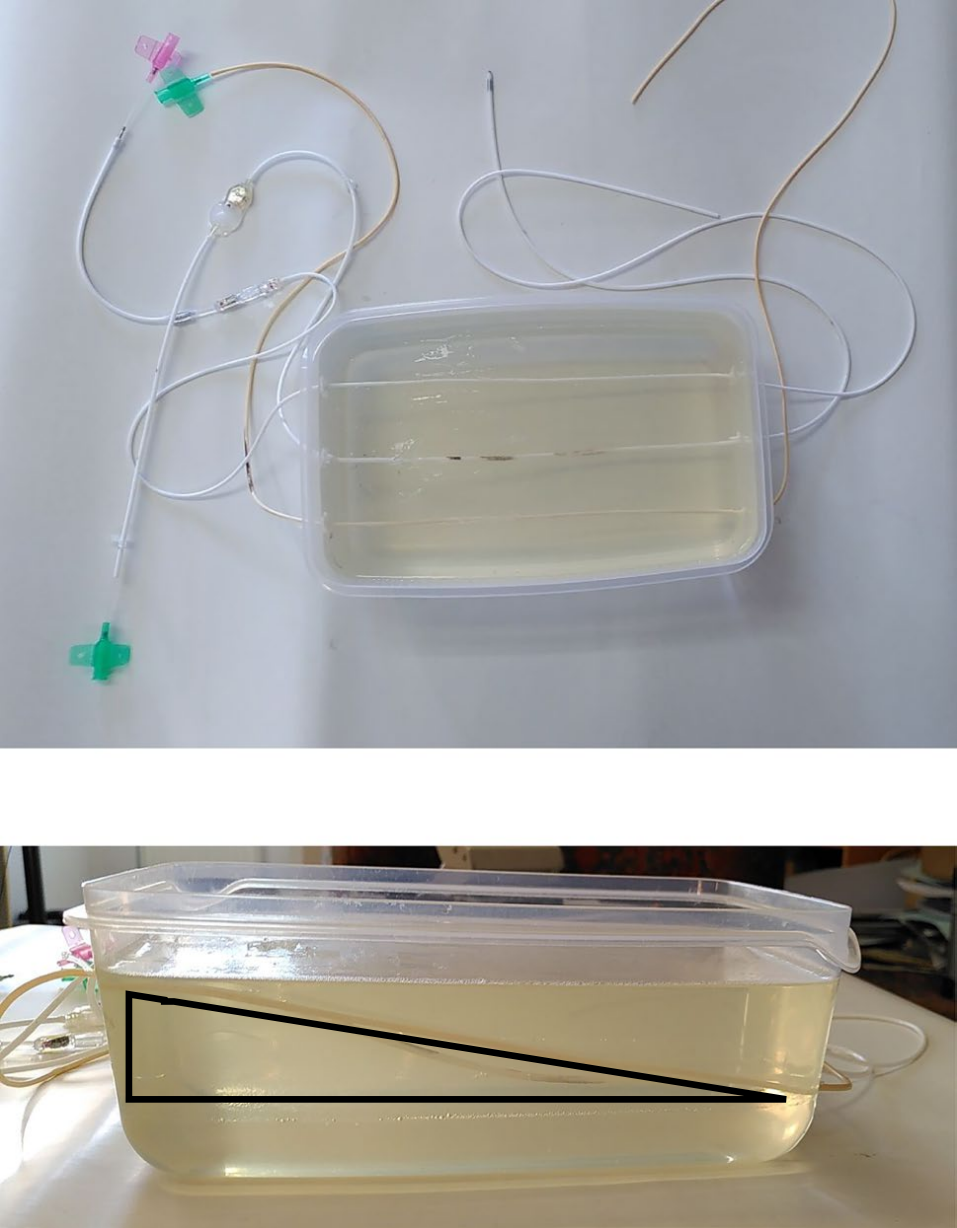
Ultrasound phantom generated with gelatine and agar–agar powder solved in water and three cerebral shunts inserted with defined slope (black triangle)

In order to allow reproducible measurements of VP shunt flow in a standard setting, we developed a phantom model composed of a polypropylene box filled with a gelatin mix containing different CSF shunt systems, which were connected to a pump system, allowing for a controllable flow of water through the shunt system (Figs. 1, 2, 3 and 4).

A Canon Aplio i800 US device (Tokyo, Japan), equipped with an 11-MHz linear array transducer, was used. A SMI standard imaging protocol was established in accordance with Canon application specialists, named “VP shunt” (Fig. 3).

The VP shunt systems were suspended in a rectangular plastic tank with a size of 15*23*8 cm (Fig. 4). Three different standard VP shunt systems were used in this setting, comprising a Codman Hakim® Programmable Valve, a Codman Bactiseal® Catheter System (Both Codman & Shurtleff Inc., Raynham USA) and a Medtronic Ventricular Catheter, standard barium impregnated (Medtronic Inc., Minneapolis USA). The peritoneal catheter lumen diameters were 1.3 mm, 1 mm, and 1 mm, respectively. The connector part had a lumen diameter of 1 mm.

For the preparation of a useful flow-phantom for ultrasonic measurements several attempts were made to get the final mixture of water, gelatin, and agar–agar. Conventional food gelatin powder (Dr. Oetker, Germany), standard agar–agar powder (Pharmacy), and tap water was used. Since different specifications for solving agar–agar in 500-mL water were found in the literature, our tests showed a quite similar gelification result when using 7.5-g agar–agar or 9-g gelatin. When using gelatin, only additional 20–25% (weight proportion) gelatin has to be considered to replace agar–agar. In our case we used a gelatin equivalent of approx. 4.8-g per 100-mL water. To avoid clumpy gel, the water was heated to 60 °C and stirred when adding the gelatin or agar–agar powder, respectively. In total, 1500-mL gel mixture were filled into a rectangular polypropylene box (15*23*8 cm, IKEA, Sweden) with 2500-mL capacitance. On both smaller sides three holes were drilled—at one side 2 cm below the top and on the opposite side 1 cm above the box’s bottom to gain a well-defined slope for the catheters inserted. After placing and sealing the catheters with silicone glue, the warm gel–water mixture was added. Storing the phantom for several hours in a refrigerator assured a proper hardness of the gel phantom. This phantom was kept in use for just a couple of days to prevent seeding by mildew.

The phantom model allowed to create a realistic diagnostic setting with appearance of the VP shunt catheter system in sonography comparable to the in vivo situation. CSF flow was simulated in this phantom setting. To standardize the flow pattern, a micro-controlled syringe pump system (Angiomat Illumena, Liebel-Flarsheim, Soma Tech Intl., 166 Highland Park Dr., Bloomfield, CT 06002, USA) with possible flow rates from 0 to 450 ml/min for intra-arterial contrast application used in the angiographic suite was filled with water. The pump system was set to a flow rate of 2 ml/min. Manual positioning of the linear US transducer was performed to scan the catheter in the phantom. Upon US imaging, the center of the catheter was depicted as hypoechoic central part, while the catheter wall appeared as hyperechoic periphery. SMI US was used to confirm or exclude the presence of a flow wave pattern within the shunt systems.

The presence of a flow wave pattern upon ultrasound confirmed a normally working shunt system. In case of absence of a flow pattern, the ultrasound probe was placed on the proximal and distal catheter in order to differentiate proximal from distal shunt obstruction.

## Results

The phantom described above, which was developed specifically for this proof of principle study, corresponds to the in vivo situation of US flow measurement in a patient with a VP shunt system, regarding the physical properties and the constituting components. The gelatin filling of the polypropylene phantom creates pressure on the VP shunt system equal to the physiologic subcutaneous pressure of 3 to 5 mmHg in normal weight individuals and 15 to 20 mmHg in patients in prone position with soft layering underneath. In addition, changes in composition of CSF can be simulated using a glucose infusion, with a standard temperature setting of 37 °C.

**Fig. 5:**
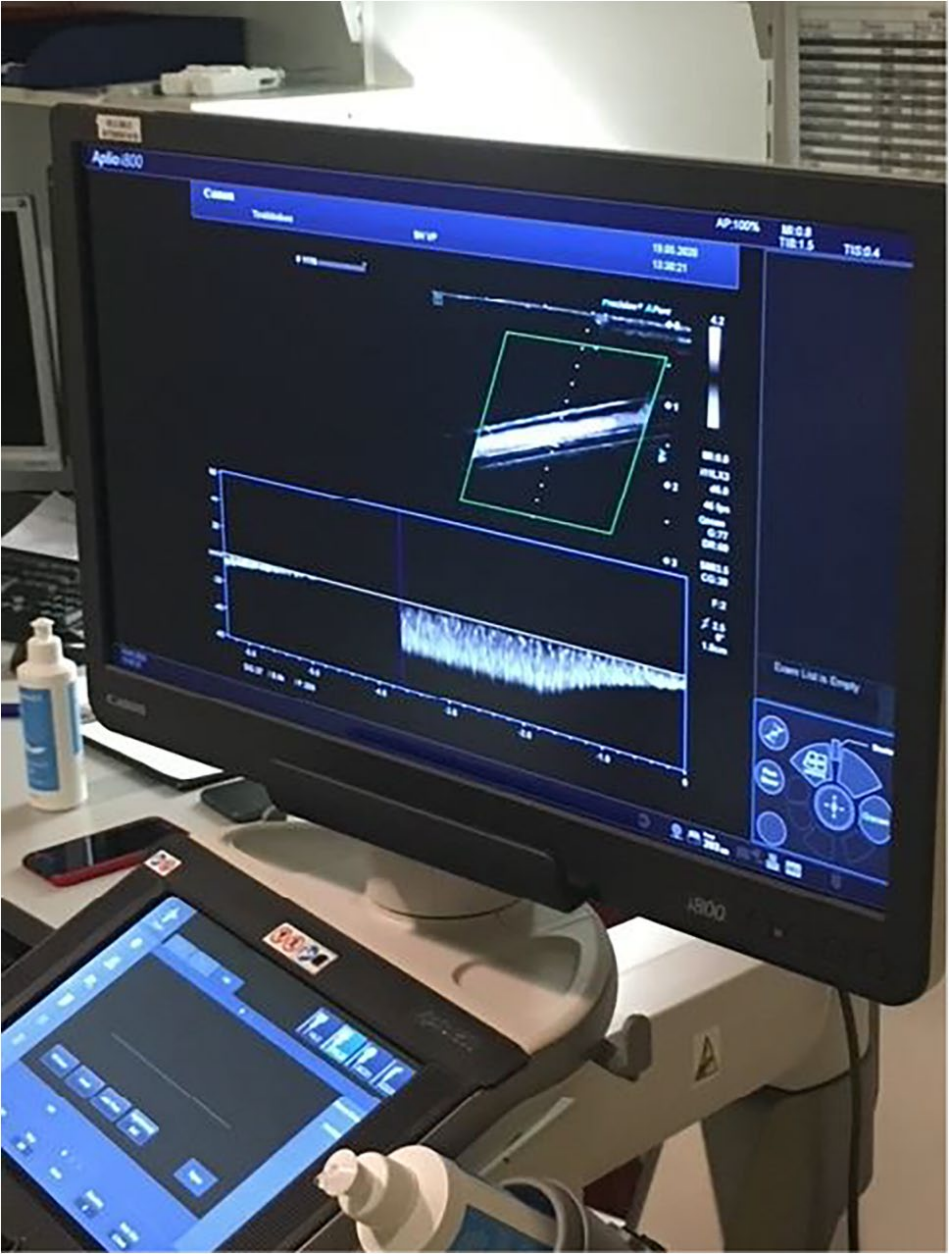
The image shows the ultrasound scan of a shunt catheter, with a clear demarcation of the inner lumen and the tube wall. The flow was created using the pressure pump system, and the SMI scan shows a continous flow specrtum through the patent shunt, which is located in the phantom

We performed repeated ultrasound measurements using the same phantom model and the same US machine. All three shunt systems were evaluated on 6 occasions, with a median expenditure of time of 15 min for evaluation of each individual shunt system in the phantom model, resulting in an investigation time of 45 min for each session. Upon US imaging, the shunt catheter appeared clearly visible with a hyperechoic outer rim and a hypoechoic central part. The presence of flow through the shunt systems was confirmed correctly by evaluation of the flow wave pattern in US using SMI. SMI wave patterns corresponded to the flow created by the pump system, regarding temporal and local correlation (Fig. 5).

**Fig. 6:**
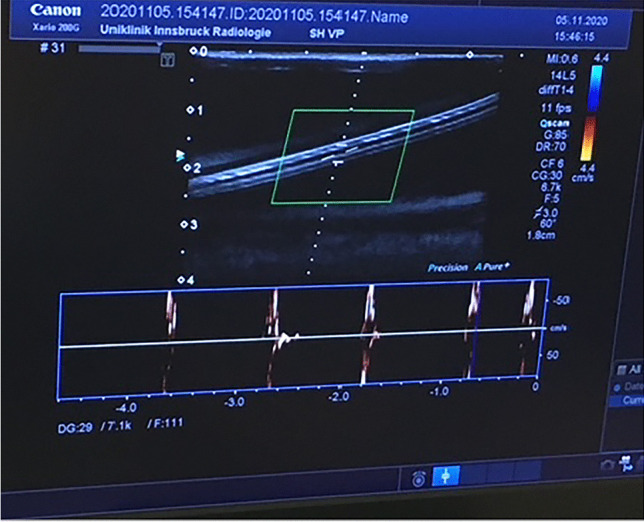
SMI scan of a shunt system in the phantom with pulsatile flow spectrum while pressure is being applied on the reservoir of the valve, which is an integral part of the shunt system, manually

This was evaluated by activating the pump system and again suspending the system in order to confirm presence and absence of flow wave patterns in SMI US, respectively. Additionally, if the valve in the reservoir of the CSF shunt system was pressed, the resulting flow was directed towards the distal part of the catheter (Fig. 6 and ESM Fig. 7).

The visibility of a flow wave pattern in SMI imaging correlated directly to the flow of water through the catheter system and was absent if the pump system was interrupted. For the confirmation of the presence or absence of flow wave patterns, only qualitative measurements were necessary, not necessitating further quantitative measurement of flow rates.

Further evaluation of the localization of shunt malfunction was feasible in this phantom setting, as it included the ventricular part of the shunt system, the valve, and the peritoneal part of the shunt system. Mechanical pressure on the reservoir of the valve resulted in a reproducible flow wave pattern, distally.

However, if the catheter was obstructed at the exit port, no flow wave pattern was detectable along the peritoneal part of the shunt catheter in SMI ultrasound. Consequently, if the catheter valve was pressed in this setting, a retrograde wave pattern was observed in the ventricular part, and once again when the pressure on the valve was released. On the other hand, if the ventricular end of the catheter was obstructed, no flow wave patterns were observed on both sides of the valve.

## Discussion

The evaluation of possible acute VP shunt dysfunction poses a significant challenge in clinical routine, despite technical advances in clinical imaging. Clinicians have to evaluate whether a VP shunt system is obstructed or not in symptomatic patients immediately to decide whether surgical revision of the VP shunt system is indicated or not, in order to avoid severe complications and potential fatality. Additionally, the localization of the obstruction site is a further diagnostic challenge in clinically symptomatic VP shunt patients.

Previous studies evaluated the role of Duplex US for the evaluation of shunt patency [9]. Although this noninvasive approach is promising, it is of limited use in a daily clinical routine setting, as no standards are available regarding the regions of measurement and the possibly low flow rates with expectably significant variation in flow velocity. Up to now, there have been several proposals for the use of US in the evaluation of patency of CSF shunt systems. Hartman et al. proposed the use of contrast enhanced ultrasound (CEUS) to evaluate the flow in patients with ventriculoperitoneal shunt systems [10]. However, the application of microbubbles in the venous system is a more invasive procedure and more costly in comparison to SMI. This technique requires an ultrasound machine being equipped with a specific software package for visualization of the contrast media. Further investigations are needed to compare the accuracy of different US techniques in the clinical setting. Furthermore, the role of US in the evaluation of VP shunt system dysfunction has been compared directly to the application of radioactively labeled substances, performing radionuclide imaging of ventriculoperitoneal shunt systems [11, 12]. However, radionuclide imaging exposes the patient to a significant radiation burden and requires nuclear medicine facilities, which are not readily available. In addition, nuclear medicine imaging requires more time than an US investigation. Furthermore, radionuclide imaging has a limited technical resolution as well as limited resolution regarding the specificity of the tracer distribution and the minimal detectable amount of radiation.

The ease of application of the US examination and the low expenditure of time for the procedure make SMI US an attractive approach for the evaluation of patency of ventriculoperitoneal shunt systems in symptomatic patients. SMI facilitates the detection of CSF flow even in VP shunt systems with very low flow rates. After manual squeezing of the valve, which is an integral part of the most VP shunt systems, the evaluation of patency is performed, using SMI US for confirmation of the presence or absence of flow wave patterns. The presence of a flow wave pattern in the VP shunt system in this setting confirms patency. However, due to variations in flow rates, a definition of standard values for CSF flow rate values remains challenging.

Moreover, the SMI US approach bears the potential to identify the site of shunt obstruction effectively. If the distal part of the peritoneal catheter is obstructed, the valve is emptied upon pressure and refills again, which can be observed directly in SMI US imaging, while SMI evaluation reveals the absence of a wave pattern in the same patient in the resting situation. If flow wave patterns are assessed in the ventricular part of the catheter, patency can be directly confirmed by SMI US, showing a corresponding US flow wave pattern. In case of obstruction of the ventricular part of the catheter, no US flow wave pattern can be observed directly there.

Nevertheless, our study has some limitations, including the artificial setting of a phantom model, making direct comparison to in vivo situation difficult.

Translation from the phantom model into the clinical situation of VP shunt dysfunction may bear some difficulties, as the SMI US measurement is dependent on the recording site. First of all, identification and localization of the ventricular and peritoneal segments of the VP shunt are mandatory. This may be hampered by skin thickness, hair coat, scar tissue, and interfering osseous structures, for example, the clavicle in the neck region. In fact, limitations in penetration depth have to be considered when using high frequency US probes. The localization of the peritoneal part may be difficult in obese patients or due to bowel gas. In particular, the localization of the segments of the VP shunt can be hampered by anatomical reasons. In addition, correct positioning of the US probe for accurate probe coupling and thus beam angulation during Duplex US depiction and US Doppler measurement may be limited as for example in patients with short necks. Duplex US measurement in fact can be performed accurately with an angulation of the sound beam of up to 60° at the VP shunt tube. If the correct positioning of the probe and an accurate angulation was not feasible, correct US evaluation of VP shunt patency was impossible due to underlying physics.

Furthermore, the pressure application by the pump system is comparable to the pressure application in vivo but may differ according to the individual clinical situation.

The uppermost advantage of SMI evaluation is that wave patterns are present independent of changes of the flow rates. Therefore, the confirmation of wave patterns in SMI US is sufficient to evaluate the VP shunt function or dysfunction during clinical routine, reducing the expenditure of time for evaluation of symptomatic VP shunt patients and leading to a clear and early decision on a necessity of surgical revision by application of a modality, which is generally readily available.

## Conclusion

Even though CSF shunt systems represent the standard in the treatment of patients suffering from hydrocephalus, currently, there is no readily available, established standard imaging procedure to evaluate shunt system dysfunction in an acute setting. In this study, we conducted a test on a CSF shunt flow measuring system using SMI ultrasound in order to confirm or exclude patent flow through different VP shunt system in a standardized phantom setting. In this model, the flow rates were expected to range between 0.05 and 30 ml/min. SMI ultrasound is a highly sensitive and cost-effective imaging modality to safely evaluate patients with CSF shunt system dysfunction and patency effectively.

### Supplementary Information

Below is the link to the electronic supplementary material.

Supplementary file1 (MOV 27662 KB)
